# Diagnose auf den zweiten Blick: Retropharyngeales Hämatom nach Sturz unter oraler Antikoagulation

**DOI:** 10.1007/s00063-020-00751-7

**Published:** 2020-10-15

**Authors:** L. Marck, B. Yuen

**Affiliations:** 1 Institut für Intensivmedizin, Universitätsspital Zürich, 8091 Zürich, Schweiz; 2 Interdisziplinäre Intensivstation, Spital Bülach, 8180 Bülach, Schweiz; 3 Universität Zürich, Zürich, Schweiz

## Anamnese

Ein 91-jähriger Patient stellte sich kurz nach häuslichem Sturz mit frontalem Kopfanprall an einem Zaun auf der Notfallstation vor. Er klagte über Kopfschmerzen ohne Schwindel, Übelkeit oder Erbrechen. Die tägliche Medikation bestand aus Pregabalin und Rivaroxaban.

## Klinischer Befund

Auf der Notfallstation präsentierte sich der Patient wach und orientiert sowie kardiopulmonal stabil. Im primären Bodycheck zeigten sich eine Prellmarke im Bereich der rechten Wange sowie eine Druckdolenz über der Halswirbelsäule, woraufhin die Immobilisierung mittels Immobilisationskragen erfolgte. Die weitere körperliche Untersuchung war unauffällig. Insbesondere der Halsbereich wies keine Schwellungen oder Prellmarken bei fehlendem Stridor und unauffälliger Sprache auf.

## Labor/Bildgebung

In der Nativ-CT von Halswirbelsäule und Schädel gab es keine Hinweise auf eine Fraktur oder eine intrakranielle Blutung. Im Labor zeigten sich eine milde Anämie (Hämoglobin 13,5 g/dl), eine Hyponatriämie (S-Natrium 134 mmol/l) und ein INR von 1,5 unter Antikoagulation mit Rivaroxaban.

## Notfallmedizinischer Verlauf

**Figure 1:**
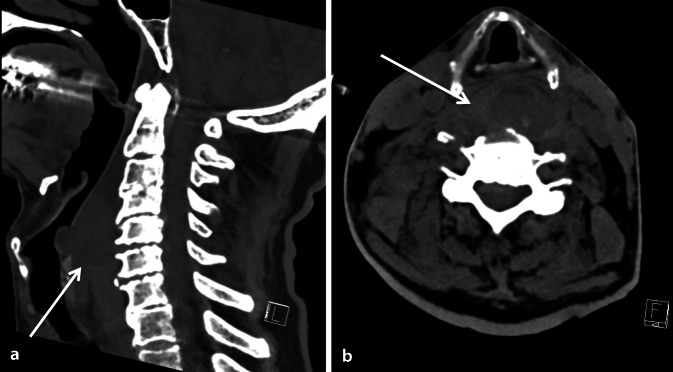

Rund sieben Stunden nach Sturzereignis entwickelte der Patient noch auf der Notfallstation neu einen unproduktiven Husten. Nach einem Hustenanfall kam es kurze Zeit später zu einem Atemstillstand mit Zyanose, gefolgt von einer hypoxämieinduzierten Bradykardie (Herzfrequenz 35/min), welche mit 0,5 mg Atropin therapiert wurde. Unter der sofort eingeleiteten Maskenbeatmung konnte rasch eine adäquate Oxygenierung gewährleistet werden. Ein konventioneller Intubationsversuch war bei nicht einzusehender Stimmbandebene und diffuser supraglottischer Weichteilschwellung erfolglos. Bei definitionsgemäß vorgelegenem „difficult airway“ erfolgte der zweite Intubationsversuch fiberoptisch, welcher gelang. Danach wurde der Patient hämodynamisch stabil und mechanisch beatmet auf die Intensivstation verlegt (Abb. 1).

## Wie lautet Ihre Diagnose?

**Diagnose:** Traumatisches retropharyngeales Hämatom unter oraler Antikoagulation

## Intensivmedizinischer Verlauf

**Figure 2:**
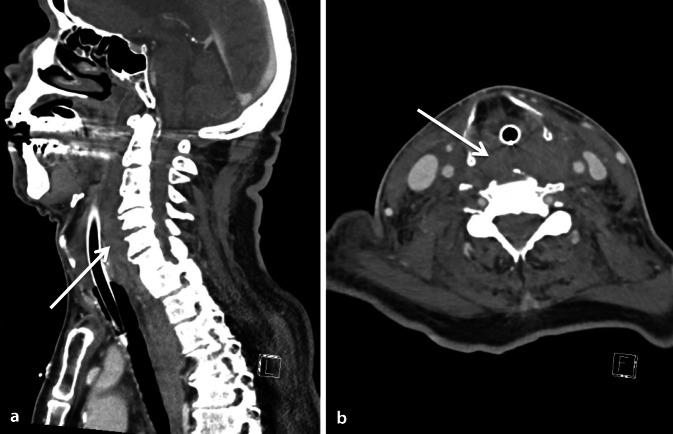

Am Folgetag demarkierte sich eine rechtsseitige Schwellung im Halsbereich, entsprechend einem Hämatom im Ultraschall. In der erneuten Durchsicht der CT-Aufnahmen vom Vortag zeigte sich mit Fokus auf die Halsweichteile eine symmetrische Weichteilschwellung mit verdrängender Tendenz der umliegenden Strukturen, vereinbar mit einer Einblutung in den Musculus longus colli (maximale Breite 25 mm) von Höhe HWK 3 bis HWK 7. Außerdem zeigte sich im Seitenvergleich eine verdickte Skalenusmuskulatur rechts und eine Verlagerung der Trachea nach ventral, passend zu einem retropharyngealen Hämatom. Zudem konnte eine Fraktur des Tuberculum caroticum anterior des sechsten Halswirbelkörpers festgestellt werden. Nachdem die Bedside-sonographischen Kontrollen stets einen stationären Befund ergaben, zeigte die kontrastmittelverstärkte CT-Verlaufsbildgebung am Folgetag bereits eine Regredienz des Hämatoms des Musculus longus colli. Eine aktive Blutung konnte dabei ausgeschlossen werden. Angesichts des rückläufigen Befunds war eine operative Entlastung gemäß Rücksprache mit den Chirurgen nicht indiziert, sodass der Patient bei unauffälligem Cuff-Leck-Test und suffizienter Spontanatmung nach 36 h komplikationslos extubiert werden konnte. Aufgrund steigender Entzündungsparameter und pulmonaler Infiltrate im konventionellen Thoraxröntgenbild wurde eine empirische antibiotische Therapie mit Amoxicillin/Clavulansäure verordnet. Auf die Gabe von Kortikosteroiden haben wir bei fehlender Evidenz verzichtet. Im Verlauf wies der Patient einen zunehmend hohen Sauerstoffbedarf auf (p_a_O_2_/F_i_O_2_ 83 mm Hg unter High-flow-Sauerstofftherapie). Im Thoraxröntgenbild zeigten sich progrediente Infiltrate beidseits, passend zum Bild eines „acute respiratory distress syndrom“ (ARDS) in Folge einer Aspiration. Hinweise auf eine erneute Atemwegsverlegung gab es keine. Eine Reintubation wurde vom Patienten abgelehnt, woraufhin bei progredienter respiratorischer Insuffizienz auf eine Palliation umgestellt wurde und der Patient später auf der Intensivstation verstarb (Abb. 2).

## Definition

Ein posttraumatisches retropharyngeales Hämatom stellt selbst bei Patienten unter oraler Antikoagulation eine seltene Blutungskomplikation dar und tritt zum Teil mit hoher Latenzzeit auf [2]. Häufig fehlen zum Zeitpunkt der radiologischen Untersuchung klinische Hinweise für eine retropharyngeale Hämatombildung.

Bei entsprechendem Traumamechanismus erfolgt in der Regel eine computertomographische Bildgebung des Kopfes zum Ausschluss einer intrakraniellen Blutung, häufig ergänzt um eine Bildgebung der Halswirbelsäule zum Frakturausschluss. Die Untersuchung wird in der Regel ohne Kontrastmittel durchgeführt und dient nicht primär der Beurteilung von Weichteilstrukturen.

Der retropharyngeale Raum (Spatium retropharyngeum) besteht weitgehend aus losen Bindegewebsstrukturen und wird dorsal durch die Fascia cervicalis (Lamina praevertebralis), welche lateral dem M. longus colli aufliegt, begrenzt. Nach kaudal geht der retropharyngeale Raum in den retrotrachealen Raum über und reicht von der Schädelbasis bis auf die Höhe des vierten Brustwirbelkörpers [4]. Neben Blutungen aus den dortig verlaufenden Vertebralgefäßen kann es traumaassoziiert zu Blutungen im Bereich der Längsmuskulatur (M. longus colli) oder des Ligamentum longitudinale kommen. Im beschriebenen Fall konnte bei nicht darzustellender aktiver Blutung sowie intakten Gefäßen von einer Blutung im Bereich des M. longus colli ausgegangen werden. Neben einer posttraumatischen Blutungskomplikation (Hyperextensionstrauma) stellen Katheterinterventionen im Halsbereich oder Magensondeneinlagen mögliche Ursachen einer retropharyngealen Hämatombildung dar [1]. Auch spontan entstandene retropharyngeale Hämatome wurden beschrieben [5]. Eine orale Antikoagulation begünstigt in jedem Fall die Ausbildung eines Hämatoms. Die anatomischen Gegebenheiten bedingen die in der Literatur häufig beschriebene klinische „Capps triad“, die neben Ekchymosen im Bereich von Thorax oder Hals eine Verlagerung der Trachea und Kompression von Ösophagus und/oder Trachea umfasst [3]. Bildgebend bestehen zum Teil nur indirekte Hinweise für ein Hämatom, während klinisch neben einer Darstellung bei der enoralen Inspektion Husten, Stridor, Heiserkeit und Dysphagie als mögliche Frühzeichen vorliegen können [2].

## Therapie und Verlauf

Wie im beschriebenen Fall ist eine Schwellung von außen nicht zwangsläufig ersichtlich. Therapeutisch ist eine frühzeitige Sicherung der Atemwege mittels endotrachealer Intubation (bevorzugt fiberoptisch wache Intubation) oder Tracheostomie essenziell, um eine potenziell lebensbedrohliche Atemwegsverlegung zu vermeiden. Neben einer Gerinnungsoptimierung bei Patienten mit einer Koagulopathie muss je nach ursächlicher Konstellation eine Angiographie mit Katheterembolisation bei aktiver Blutung oder eine chirurgische Exploration zur Blutstillung und Hämatomausräumung evaluiert werden [2]. Ob der Patient von einer häufig verschriebenen Antibiotika- oder Kortikosteroidtherapie profitiert, ist unklar.

Aufgrund der potenziell mehrstündigen Latenzzeit bis zum Einsetzen von Symptomen einer Atemwegsverlegung empfiehlt sich neben der stationären Überwachung von Vigilanz und Vitalparametern die Sensibilisierung gegenüber möglichen Frühsymptomen einer retropharyngealen Hämatombildung. Außerdem sollte zur Früherkennung bei der radiologischen Bildgebung stets die Beurteilung der Halsweichteile sowie des retropharyngealen Raums erfolgen und eine wiederholte Bildgebung bzw. eine fiberoptische Pharyngolaryngoskopie durch einen HNO-Facharzt bei entsprechendem Verdacht in Betracht gezogen werden.

## Fazit für die Praxis

- Zu den Frühzeichen einer retropharyngealen Hämatombildung zählen Dyspnoe, Stridor, Dysphagie und Heiserkeit. Symptome können mit einer Latenzzeit von bis zu 20 h auftreten, weshalb eine Überwachung empfehlenswert ist.
- Bei der Bildgebung sollte stets eine Beurteilung der Halsweichteile erfolgen.
- Bei einem retropharyngealen Hämatom muss eine Atemwegssicherung mittels Intubation oder Tracheostomie frühzeitig evaluiert werden.
